# Evaluation of the feasibility and acceptability of ReWin—A digital therapeutic rehabilitation innovation for people with stroke-related disabilities in India

**DOI:** 10.3389/fneur.2022.936787

**Published:** 2023-01-12

**Authors:** Sureshkumar Kamalakannan, Vijay Karunakaran, Ashwin Balaji, Aadi Sai Vijaykaran, Sivakumar Ramachandran, Ramakumar Nagarajan

**Affiliations:** ^1^Department of Social Work, Education and Community-Wellbeing, Northumbria University, Newcastle upon Tyne, United Kingdom; ^2^InGage Technologies Pvt., Ltd., Chennai, Tamil Nadu, India; ^3^Faculty of Physiotherapy, Sri Ramachandra Institute of Higher Education and Research, Chennai, Tamil Nadu, India; ^4^Department of Neurological Rehabilitation, Chennai Advanced Rehabilitation Centre, Chennai, Tamil Nadu, India

**Keywords:** stroke, telerehabilitation, health technology, mHealth (mobile Health), India, disability, continuum of care

## Abstract

**Background:**

Developing culturally appropriate, scalable interventions to meet the growing needs for stroke rehabilitation is a significant problem of public health concern. Therefore, systematic development and evaluation of a scalable, inclusive, technology-driven solution for community-based stroke care are of immense public health importance in India. ReWin is a digital therapeutics platform that was developed systematically. This study aimed to evaluate its feasibility and acceptability in an Indian context.

**Objectives:**

Phase–1: To pilot the intervention for identifying operational issues and finalize the intervention. Phase–2: To assess the feasibility and acceptability of ReWin intervention in an Indian context.

**Methods:**

Design: Mixed-methods research design. Setting: Participant's home and rehabilitation centers. Participants were selected from rehabilitation centers in South India. Participants: Ten stroke survivors and their caregivers, as well as four rehabilitation service providers were recruited for phase 1. Thirty stroke survivors who were treated and discharged from the hospital, and their caregivers as well as 10 rehabilitation service providers were recruited for Phase 2. Intervention: ReWin a digital therapeutic platform with the provider and patient app for the rehabilitation of physical disabilities following stroke was piloted. Process: Evaluation of the intervention was completed in two phases. In the first phase, the preliminary intervention was field-tested with 10 stroke survivors and four rehabilitation service providers for 2 weeks. In the second phase, the finalized intervention was provided to a further 30 stroke survivors to be used in their homes with support from their carers as well as to 10 rehabilitation service providers for 4 weeks. Outcome measures: Primary outcomes: (1) operational difficulties in using the ReWin intervention; (2) feasibility and acceptability of the ReWin intervention in an Indian setting.

**Results:**

Field-testing identified operational difficulties related to 1. Therapeutic content; 2. Format; 3. Navigation; 4. Connectivity, 5. Video-streaming, 6. Language; and 7. Comprehensibility of the animated content. The intervention was reviewed, revised and finalized before pilot testing. Findings from the pilot testing showed that the ReWin intervention was feasible and acceptable. About 76% of the participants had used ReWin for more than half of the intervention period of 4 weeks. Ninety percentage of the stroke care providers and about 60% of the stroke survivors and caregivers felt that the content of ReWin was very relevant to the needs of the stroke survivors. Forty percentage of the stroke survivors and caregivers rated ReWin intervention as excellent. Another 45% of the stroke survivors and caregivers as well as 90% of the stroke care providers rated ReWin intervention as very good based on its overall credibility, usability, and user-friendliness.

**Conclusions:**

ReWin has all the essential components to connect care providers and consumers not just for stroke rehabilitation but for several other health conditions with the use of several other technological features that support rehabilitation of persons with disabilities and strengthen rehabilitation in health systems worldwide. It is critical to amalgamate ReWin and other evidence-based interventions for rehabilitation to innovate scalable solutions and promote universal health coverage for stroke care worldwide.

## Background

Stroke is a leading cause of death and disability in India (1). Systematic review of the epidemiology of stroke in India reveals a crude stroke prevalence between 26 and 757/100,000 people per year (1). These estimates are higher than in many high-income countries (1). The effects of stroke lead to various types of impairments, such as physical, cognitive, communication, and psychological impairments (2). These impairments subsequently cause activity limitations in daily life and restrict an individual from participating in individual, family, and social roles (2). These disabling experiences of stroke profoundly impact the quality of life and limit the participation of stroke survivors in the society and economy (3). It also implies substantial rehabilitation needs that could be experienced by the stroke survivors and their caregivers who are usually the family members in an Indian context (4).

However, access to comprehensive multi-disciplinary stroke rehabilitation services is very limited in an Indian context. Rehabilitation services to persons with disabilities, in general, are hardly available. Even if they are available, they are restricted to private hospitals located in urban cities (5). Given this situation, rehabilitation services for stroke survivors especially after their hospital discharge can only be dreamt (5). In the absence of any community-based rehabilitation services, the need for rehabilitation and the burden of care exponentially increases when stroke survivors are discharged from hospitals after acute care and when they start living in their communities (6). The burden of care on the family members (informal caregivers) who lack knowledge and skills for this role could have devastating consequences for the stroke survivors during their road to recovery following a stroke (6). Close to 50% of those who survive a stroke endure post-stroke depression in India (7). Additionally, the current COVID-19 pandemic had exponentially increased these rehabilitation needs as well as the burden of the caregivers of persons with disabilities, particularly stroke survivors worldwide (8).

Developing a culturally appropriate, scalable interventions to meet these growing needs for stroke rehabilitation is a significant problem of public health concern. Given the resource limitations, alternate strategies driven by technology which is a surplus in India need to be considered (9). Technology-driven educational interventions for stroke care have been developed and proven to be feasible and acceptable in India (10). However, such standalone interventions have not proven to be effective clinically and also for health system strengthening (11). This implies the need to develop scalable solutions, which are community-based and that include stroke survivors, their caregivers, rehabilitation experts, stroke service providers, and the overall system for stroke care (12). Therefore, systematic development and evaluation of a scalable, inclusive, technology-driven solution for community-based stroke care are of immense public health importance in India.

**Figure 1:**
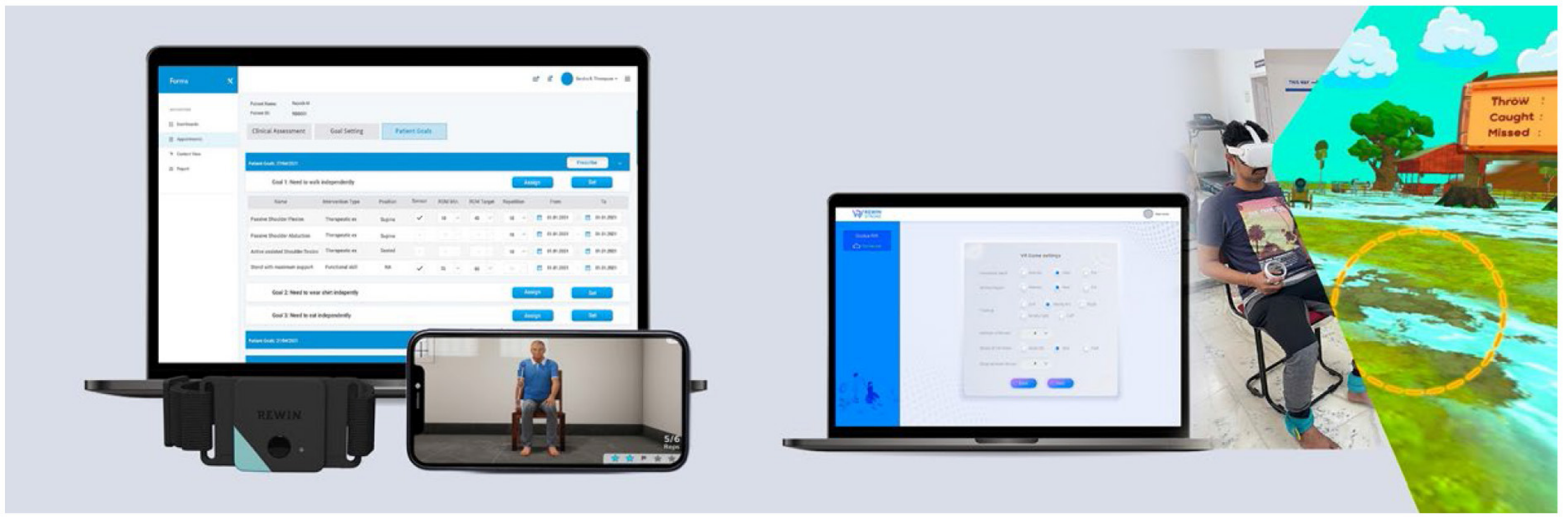
The ReWin intervention.

ReWin is a technology-driven solution developed to meet the needs of people with disabilities in India. The intervention connects stroke survivors and caregivers at home and the stroke care providers in hospitals through an application that is designed and includes therapeutic information in video format, therapeutic assessment, and rehabilitation through biofeedback sensors and virtual reality, respectively (Figure 1). ReWin is a digital therapeutics platform conceptualized, developed, and owned by InGage Technologies, a company based out of Chennai, India (13). The intervention was developed systematically based on the recommendations of the Medical Research Council UK (14). As a next step, it was evaluated for its feasibility and acceptability in an Indian context.

## Objectives

The study was conducted in two phases. The objectives are the following:

### Phase–1

To pilot the intervention for identifying operational issues and finalize the intervention.

### Phase–2

To assess the feasibility and acceptability of ReWin intervention in an Indian context.

## Methods

### Study design

It was a mixed-methods study that was carried out in two phases (15).

#### Detailed methods for phase–1

The objective of this phase was to identify operational issues of the draft Rewin Intervention to ensure its fit for purpose. We assessed the ability of the expert to access the intervention and initiate virtual assessment, rehabilitation, and follow-up using ReWin. We also assessed the ability of stroke survivors and their carers to operate a smartphone for accessing the ReWin intervention and engage in home-based therapy and care. Additionally, we observed the participants while they used the intervention to determine the training needs and operational requirements related to the intervention. Findings from each of the activities within the phase were used to triangulate information and support our synthesis of findings (15).

**Study Setting:** Rehabilitation Centers and Communities (Home-based).

**Participants Inclusion Criteria:**

**Group–1 Stroke Survivors:**

- Patients with recent stroke (diagnosed within 6 weeks) as defined by WHO.
- Adults aged ≥ 18 years.
- Presenting with Minor and Moderate stroke [i.e., scoring 1–15, according to the National Institute of Health Stroke Scale (NIHSS)] (16).
- Having a caregiver with them.

**Group–2 Providers:**

- Qualified Health care professionals with experience in providing therapy and rehabilitation services to stroke patients (physiotherapist, occupational therapist, rehab nurse, etc.).
- Willing to actively engage in follow–up of their stroke patients.

**Participants' Exclusion Criteria:**

- Stroke survivors with severe communication problems identified using the NIHSS.
- Stroke survivors who cannot provide consent autonomously.
- Those presenting with severe stroke [i.e., scoring < 15, according to the NIHSS (16)].

**Sampling:** Purposive.

**Sample Size:** Ten stroke survivors and their caregivers as well as four-stroke care professionals.

**Study Process:**

As a first step, written informed consent was obtained from all the study participants before engaging them in the study.

**Training and equipment handover:**

- The stroke care professionals, stroke survivors, and caregivers were provided training to use the Rewin Intervention by the developers.
- Following successful training (able to access and use all the components of the intervention of their specific versions at least twice without any support), the ReWin intervention was handed over to the health professionals and the stroke survivors to be used for the next 2–3 days.

**Direct observation:**

The utilization of the intervention among each group of participants was directly observed by the investigators. Issues related to training, operation, technology, access, interface, content, and processes were directly observed and documented. Please find the observation checklist enclosed as a Supplementary material.

**Interviews:**

Interviews were conducted among selected participants in both groups to understand their experiences and difficulties in using the ReWin and the issues identified during the direct observation were also triangulated. Please find the semi-structured interview guide enclosed as a Supplementary material. The reasons for the issues reported were also identified. The issues identified were rectified and the Rewin intervention was revised to fit the purpose of the study.

#### Detailed methods for phase–2

This phase applied mixed research methods to collect more comprehensive evidence and have a deeper understanding of the feasibility and acceptability of ReWin Intervention (15).

**Study Setting:** Rehabilitation Centers and Communities (Home-based).

**Criteria for inclusion:**

**Participants:**

There were three sets of participants

1. Qualified professionals (therapists) involved in therapy and rehabilitation.

2. Stroke survivors.

3. Carers of stroke survivors.

**Qualified Professionals:**

- Therapists (Physiotherapists and Occupational therapists)
- Professionally qualified undergraduates or post-graduates
- Currently practicing stroke rehabilitation.

**Stroke survivors:**

- Patients with recent stroke (diagnosed within 6 weeks) as defined by WHO.
- Adults aged ≥ 18 years
- Presenting with minor and moderate stroke (i.e., scoring 1–15, according to the NIHSS).
- Having a caregiver with them.
- Accessing rehabilitation services either as an inpatient or an out-patient.
- Medically stable (reaching a point in medical treatment where, life-threatening problems following stroke have been brought under control).
- Without severe cognitive-communication problems.
- Without severe comorbidities.
- Willing to adhere to study protocols.
- Qualify the training requirements (using ReWin on their own or with support from caregivers).

**Carers of stroke survivors:**

- Adults ≥ 18 years.
- Primarily caring for the PWDs.
- Can comprehend and communicate.
- Willing to adhere to study protocols.
- Qualify the training requirements (using ReWin on their own to support stroke survivors).

An eligibility assessment was conducted by the investigators to identify participants to be recruited for the study.

**Participants Exclusion Criteria:** It remained the same as the exclusion criteria for phase 1.

### Participant recruitment for the study

Qualified professionals for this phase of the study were identified with support from the collaborating institutions. Stroke survivors and their caregivers referred for therapy services were identified through hospital/rehabilitation center's records. Preliminary Information about participants who fulfill the eligibility criteria was retrieved from records. An eligibility assessment was completed within 2 days after identification through a telephone call followed by a home visit. Caregivers of the eligible participants were assessed for their eligibility to be recruited in the study.

All participants (stroke survivors, caregivers and stroke care providers) identified for this phase of the study were contacted and briefed about the study by the investigation team. They were informed about the purpose and processes of the study. If a participant is interested, written informed consent was obtained in person from them. Consent procedures were completed either at the health facility or at the participant's home whichever is feasible for the participants.

### The sample size for the pilot study

Sampling was purposive. The collaborating hospitals had many health professionals in their comprehensive rehabilitation system. However, we recruited 10 qualified health professionals/therapists who met our eligibility criteria for the study. The admission rate of stroke survivors who fulfilled the set criteria at the collaborating institutions was 4 per week. Given the admission rate, and the study plans. It took 4 months to recruit 30 stroke survivors and their carers who met the eligibility criteria for this phase.

### Study procedure for feasibility and acceptability assessment

ReWin had two separate applications. One for the therapists and one for the stroke survivors and their caregivers (13). The key strategy for the evaluation involved.

a. Training the study participants separately to access and use their respective apps for assessment or self-evaluation.

b. Collaboratively, setting one functional goal reflecting on activities of daily living and discussing its relevance and feasibility for achievement using ReWin (specific goal-setting features, and consultation features).

c. Prescription of therapeutic activities or exercises by therapists using ReWin App.

d. Stroke survivors and caregivers follow the prescribed therapeutic plan at their homes and follow up with the therapists for any support using ReWin.

The details of the ReWin innovation and the features mentioned above is described elsewhere (13).

### Health professionals

Qualified therapists being recruited for this study attended a 2-h group training and discussion on using ReWin in their day-day practice. The training was structured as two sessions

1. Introduction to the ReWin intervention.

2. Using the ReWin intervention to achieve desired goals.

At the end of the session, there was an assessment to ensure they are eligible for inclusion in the study. Those who deem fit to be recruited were provided with the ReWin device/App credentials for using it in their everyday practice for 4 weeks. The investigator ensured weekly telephonic or in-person follow-up with the therapists recruited for the study.

### Stroke survivors and their caregivers

Similarly, stroke survivors recruited for this study underwent training on an individual basis to use the ReWin intervention with or without support from their primary caregivers. Once they were found eligible for inclusion through the post-training assessment, they were provided with the ReWin device/App to be used at the bedside (if in-patient) or at home (if Out-patient) for 4 weeks. The ReWin intervention was handed over to them along with therapeutic advice by their therapists in charge. The therapist in charge followed up with the participants every week. Stroke survivors and caregivers were also encouraged to contact the investigator if they have any concerns during the 4 weeks of the study period.

### Experience interviews

Semi-structured interviews were conducted among all the participants recruited for the study at the end of the 4-week follow-up period.

#### Study tools

Separate questionnaires and topic guides were developed for each group of participants. The tools were piloted and revised before the study commencement. The questionnaire predominantly included closed-ended questions with scaled responses. The in-depth interviews had specific topic guides with open-ended questions.

### Direct observations

Utilization of the ReWin intervention by the providers and the stroke survivors with/without the support provided by the caregivers was assessed using direct observation techniques. The main purpose of using a direct observation technique is to triangulate and affirm the information provided by the participants during the experience interviews. Some of the key issues that will be assessed during the direct observation include:

a) Relevance and comprehensibility of the intervention.
b) Operational difficulties of the participants in using the intervention.
c) User-friendliness of the intervention.
d) Technical issues in the intervention.
e) Training needed in order to use the intervention.

#### Analysis plan for the study

For quantitative data, the distribution of frequencies and proportions were reported. STATA was used for the analysis of the data in the pilot phase. Similarly, the qualitative data were analyzed thematically using the framework approach to analysis.

### Ethics approval

This study was approved by the ethics committees of Arupadai Veedu Medical College, Pondicherry, and Sri Ramachandra Institute of Higher Education and Research (SRIHER), Tamil Nādu. The study was also registered with the clinical trial registry of India CTRI/2021/11/037894.

## Results

### Phase–1: Operational issues in the draft ReWin intervention

Direct observations and interviews among the participants of the study phase–1 helped identify specific operational issues in the ReWin intervention that needed revision. The issues identified were

1. Content—Participants requested more content related to therapeutic exercises as the draft version had more content related to information and minimal content related to home-based therapy. Some of the content for therapists were visible to the stroke survivors.

2. Format—Some of the animated contents were not humanly possible (e.g., The joint movements were out of range) for practicing. Some of the environments (background) of this animation was not reflecting an Indian home environment.

3. Navigation—Participants found it difficult to navigate between different sections using the interfaces of the application.

4. Language—The content of the intervention was in Tamil, but the dialect and accent were not colloquial.

5. Comprehensibility of the Animated content—Some of the animated content was not clearly understood and the participants found it difficult to use that for everyday practice.

6. Connectivity and streaming—Occasionally the content streaming was delayed due to connectivity issues.

**Insights from Interviews:**

Interviews with participants brought out some important aspects to consider for feasibility and acceptability. They were

1. Ensuring the content is multi-disciplinary with inputs from various rehabilitation professionals involved in the rehabilitation of persons with disabilities.
2. ReWin intervention can include more therapeutic content with additional technological features that enhance the virtual reality experience and data generation through automated report generation.
3. Enhancing the user-friendliness of ReWin by incorporating administrative aspects for booking appointments, therapy goal consultation, progress monitoring, and continued follow-up.

### Phase–2: Feasibility and acceptability of the ReWin intervention

Thirty stroke survivors, their caregivers, and 10 stroke rehabilitation providers from two collaborating institutions participated in the pilot study to evaluate the feasibility and acceptability of ReWin. Participants in this phase used the ReWin intervention that was redesigned and revised based on the findings from Phase–1. The details of the training provided to the participants and their performance for recruitment in the study are provided in Tables 1, 2.

**Table 1:** Details of training for therapists.

**Details of training for therapists**				
**S. no**	**Training topic**	**Mode of delivery**	**Training duration in hours**	**Number of** **attempts to qualify**
1	Introduction to ReWin	Face to face	1	3
2	NIHSS training	Online	2	4
3	App orientation	Face to face	1	3
4	Sensor orientation	Face to face	1	4
5	Virtual reality orientation	Face to face	3	4
6	Therapist app clinical training	Face to face	3	3
7	Therapist app administrative training	Face to face	2	4

**Table 2:** Details of training for stroke survivors and caregivers.

**Details of training for stroke survivors and caregivers:**				
**S. no**	**Training topic**	**Mode of delivery**	**Training duration in hours**	**Number of** **attempts to qualify**
1	Introduction to ReWin	Face to face	1	4
2	Barthel index	Face to face	2	5
3	App orientation	Face to face	2	5
4	Sensor orientation	Face to face	2	5
5	Virtual reality orientation	Face to face	2	6
6	Patient app training for self-assessment, goal setting, and performance monitoring	Face to face	4	7
7	Patient app communication and administrative training	Face to face	3	5

The ReWin intervention had two separate applications. A therapist App and a Patient App. The service providers used the therapists' App, and the stroke survivors and caregivers used the patient App. The revised ReWin intervention also incorporated two additional therapeutic components 1. The virtual reality component could enhance motor learning and manage pain. 2. Movement tracking sensors to track and provide instantaneous feedback to stroke survivors. Twelve stroke survivors used the video intervention exclusively, eight participants who had goals related to relearning movements used the video intervention and sensors together and 10 participants who had goals related to pain management and muscle strength used video intervention combined with virtual reality.

#### Relevance and comprehensibility of the ReWin intervention

All the study participants felt that the information in the ReWin intervention was presented in a way that they can watch, understand, and practice. Those who used the therapist's app reported that ReWin could have many more videos and environments for virtual reality related to exercises. About 57% of the stroke survivors and caregivers expressed that they liked the home-based exercises, and 33% liked virtual reality games. Nearly 26% of them expressed that they liked all the content. Ninety-percentage of the stroke care providers and about 60% of the stroke survivors and caregivers felt that the content of ReWin was very relevant to their needs.

#### Operational difficulties in using the ReWin intervention

All the stroke care providers (100%) accessed the ReWin intervention on their own and during the feasibility study. However, only 53% of the stroke survivors and caregivers reported that they used the video intervention on their own. The remaining 47% were primarily dependent on the caregivers for sensors and virtual reality, only 5–10% were independently accessing it. The rest of the participants were finding it difficult to access ReWin on their own and were dependent on the therapists majorly to use both the virtual reality and therapeutic sensors. About 6–7% of the stroke survivors reported that the App was slow and hence it was difficult to access the intervention.

#### User friendliness of the intervention

Stroke survivors and caregivers expressed that the ReWin intervention was very novel and impressive. They felt confident that using this intervention could enable recovery and will help in getting back to their daily life. Few participants felt they can only comment on the first impression after trying it out but felt that this ReWin could be provided at the earliest possible opportunity in acute care.

“It is really good, the best way for home care”—Stroke survivor's perspective.

Given that most of the rehabilitation centers are uni-disciplinary and predominantly led by neurologists in India, participants who used the ReWin therapist's app were all physiotherapists. Their first impression was the need for such intervention to enable a continuum of care for stroke survivors during the COVID-19 pandemic. Stroke care providers felt that it has always been difficult to follow up with stroke survivors after they are discharged but they felt ReWin has the potential to ensure continued care by connecting therapists to stroke survivors through its innovative technology. This group of participants also felt that this intervention can also result in optimal functional recovery if it was skilfully implemented.

“The three kinds of interventions can be very helpful for Patients coming here. This kind of therapy is expected to give optimal functional Recovery”—a Stroke care provider's perspective.

“We don't get to see the patients after they get discharged, so no follow-up whatsoever from our side – we feel bad about it. But ReWin helps connect with those who are discharged and supports us to provide continued care”—a Stroke care provider's perspective.

#### Training needs for using ReWin intervention

**Figure 2:**
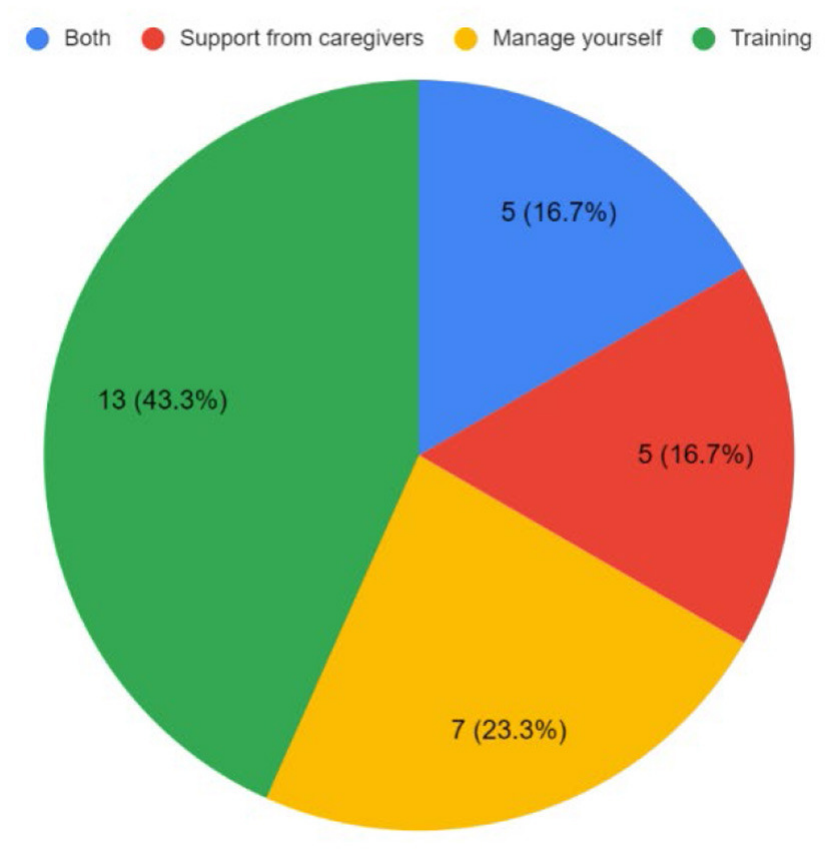
Training needs of the stroke survivors and caregivers.

Nearly 80% of the stroke care providers felt that they needed training and support to optimally utilize ReWin. Close to 23% of the stroke survivors felt they do not require any training. However, about 75% of them said they required training (43.3%), caregiver support (16.7%), and both (16.7%). Please find Figure 2. Almost all the stroke care providers felt that the training was sufficient with adequate instructions, demonstrations, and opportunities to practice ReWin. They also felt that a booklet would be very helpful to understand the methods to use ReWin in everyday practice effectively. The stroke survivors and caregivers also perceived the training as the care providers. However, 60% of them felt they do not require a booklet and about 5% felt they needed more time to practice. Overall, 90% of the stroke care providers and 55% of the stroke survivors and caregivers felt they received sufficient training and support to access ReWin. Forty-five percentage of the stroke survivors and caregivers expressed that they required more time and opportunity to practice ReWin.

#### Utilization and feasibility of the ReWin intervention

**Figure 3:**
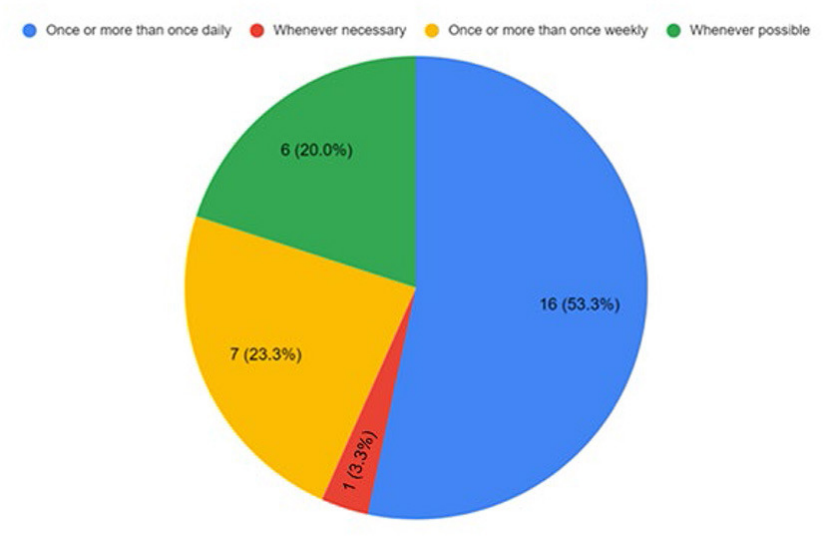
Utilization of the ReWin intervention by participants at home.

Back-end analysis of the usage of the ReWin intervention by the participants during the pilot phase revealed that 76% of the participants had used ReWin for more than half of the intervention period of 2–4 weeks. About 14% of the participants utilized ReWin between a third to half of the intervention period. The response from the stroke survivors and caregivers were also very similar to this question in the experience survey. About 53% of this group of participants reported that they used ReWin once or more than once daily and 23% of them used ReWin once or more than once weekly (Figure 3). Nearly 76% of the stroke survivors and caregivers felt that the ReWin intervention was provided to them as soon as they thought it was necessary. The responses from the stroke care providers on this were also very similar. None of the stroke survivors and caregivers have seen an intervention like ReWin before. Only 10% of the stroke care providers reported that they have seen interventions like ReWin before. Fifty percentage of the stroke survivors and caregivers expressed that they would like to use ReWin intervention for some more time. The stroke care providers too expressed the same. They expressed that they will be supporting stroke survivors and caregivers to use ReWin intervention if provided with the ReWin application.

#### Satisfaction and acceptability

About 66.7 % of the stroke survivors and caregivers expressed that they liked the intervention with utmost certainty and 33% liked the intervention to a great extent. Close to 50% of the stroke care providers expressed that they liked ReWin intervention to a great extent and the remaining proportion reported that they (definitely) liked the ReWin intervention with utmost certainty. About 80% of the stroke care providers rated the ReWin intervention as extremely useful. Similarly, 77% of the stroke survivors and caregivers rated ReWin as very useful. Close to 75% of the participants in both groups expressed that the ReWin intervention will be useful for any stroke survivors and caregivers, and they would recommend the ReWin intervention to other stroke survivors, caregivers as well as stroke care providers. Forty percentage of the stroke survivors and caregivers rated ReWin intervention as excellent. Another 45% of the stroke survivors and caregivers as well 90% of the stroke care providers rated ReWin intervention as very good based on its overall credibility, usability, and user-friendliness.

Responses of the stroke survivors and caregivers about their experience of using ReWin very clearly showed that the intervention was very useful to enable them to manage their disabilities in their homes. Most of the participants felt that they were confident to practice home-based exercises properly as prescribed by the stroke care providers and were thoroughly informed to practice with enthusiasm. The three key aspects they liked about the intervention were a technology that could connect them to stroke care providers at hospitals, interactive innovations using virtual reality and sensor biofeedback, and gamification of the exercises. Similarly, the stroke care providers felt that the ReWin intervention enables a continuum of care by ensuring regular active and passive follow-up through back-end technology. They expressed those interventions like ReWin could transform rehabilitation in resource-poor contexts and bridge the gaps in access to community-based rehabilitation. They felt technology makes it interesting to practice rehabilitation in the communities and enables them to monitor the quality of care and its impact on the lives of stroke survivors through technology-driven intervention.

## Discussion

Results from this evaluation clearly showed that ReWin intervention is feasible and acceptable to both stroke survivors, their caregivers, and stroke care providers in an Indian context. The experiences of the participants certainly reveal the need for an intervention like ReWin in contexts that have limited resources for rehabilitation, particularly in the communities. There were a couple of key concerns related to the volume of the content from the stroke care providers and the amount of time that the stroke survivors and caregivers were given to use the intervention. However, these concerns imply that ReWin is feasible for implementation, and the consumers who accessed ReWin were satisfied enough to access this intervention for an additional period. The results also revealed the confidence and motivation that ReWin instilled among the users through its design, technological intricacies, and the richness of its scientific content. The ReWin intervention was highly feasible and well-appreciated and accepted by both consumers and providers of stroke care. Similar innovations targeting rehabilitation education and information provision have been evaluated in an Indian context and it has been found feasible for implementation. However, these innovations lacked comprehensiveness. It was either aimed at a specific impairment or a specific strategy (17–19).

It is also important to note that the experience of those participants who utilized different kinds of interventions such as videos, virtual reality, and sensor-based exercises differed. Stroke care providers' support was most required for sensor-based therapy. This is especially because stroke survivors and caregivers have very minimal awareness about the placement of the sensors for specific exercise training. Similarly, stroke survivors required support from their caregivers for video-based interventions. Virtual reality was the most enjoyable aspect of the ReWin intervention however, participants felt that could be many more gamification and gamified tasks for functional and ADL training specifically. Given that wearables and sensors were expensive and need to be imported. There is a huge implication to develop low-cost solutions within India to enhance access for anyone who requires technology-driven rehabilitation. A potential strategy could be bringing together all kinds of innovations together for a comprehensive tele-neurorehabilitation system for stroke care (20).

ReWin is an innovation that can be adapted to any health condition that results in musculoskeletal impairments and subsequently any kind of functional disability. There is also a potential opportunity to expand and improvise this innovation with other kinds of impairments such as communication, psychological, and cognitive-perceptual deficits that could lead to activity limitations and participation restrictions. Consequently, there is also immense scope for translating this innovation to different languages and systematically modifying/adapting/re-inventing it to different cultures, and countries, especially in contexts where the demand for rehabilitation services is increasing and the needs are unmet. These implications from ReWin can help bridge the gaps in access to rehabilitation services not just in contexts like India but worldwide.

Given the pragmatic approach to the design of this study, limitations are inevitable. First and foremost is the use of tools that are specifically developed for the purpose of this study as opposed to standardized tools. Since we had both service providers and the stroke survivors and their caregivers as participants, it was difficult to standardize the approach to the collection of the data (naturalistic vs. controlled) from two different kinds of participants. However, the focus of the evaluation was the feasibility and acceptability of using ReWin innovation and therefore careful consideration was given to the specific issues that all kinds of participants might experience in participants' own environment (home and rehabilitation center) during the evaluation. The study used a purposive sampling strategy with a smaller number of participants. This reduces the generalizability of our findings, and the authors aim to conduct a large-scale evaluation in the potential future targeting the clinical as well as cost-effectiveness.

## Conclusion

To the best of our knowledge, interventions such as ReWin were never developed and evaluated systematically in India as well as in similar contexts. However, it does not mean that ReWin cannot revolutionize the continuum of care in HICs. ReWin has all the essential components to connect care providers and consumers not just for stroke rehabilitation but for several other health conditions with the use of several other technological features that support rehabilitation of persons with disabilities and strengthen rehabilitation in health systems worldwide. It is critical to amalgamate ReWin and other evidence-based interventions for rehabilitation to innovate scalable solutions and promote universal health coverage for stroke care worldwide. As this could revolutionize the concept of remote rehabilitation as well as enable strategizing implementation of such scalable solutions for empowering persons with disabilities.

## Data availability statement

The raw data supporting the conclusions of this article will be made available by the authors, without undue reservation.

## Ethics statement

This study was approved by the Ethics Committees of Arupadai Veedu Medical College, Pondicherry, and Sri Ramachandra Institute of Higher Education and Research (SRIHER), Tamil Nadu. The study was also registered with the clinical trial registry of India CTRI/2021/11/037894. The patients/participants provided their written informed consent to participate in this study.

## Author contributions

SK designed the study. SR, RN, and AB supported with the recruitment of participants and data collection. The manuscript was developed primarily by SK and supported by SR, AB, and VK. All authors were involved in the conceptualization of this study and reviewed and provided feedback on the manuscript. All authors contributed to the article and approved the submitted version.
