# Supplementary material for: Evaluation of the feasibility and acceptability of ReWin—A digital therapeutic rehabilitation innovation for people with stroke-related disabilities in India
Source: Front Neurol. 2023 Jan 12;13:936787. doi: 10.3389/fneur.2022.936787 (PMC9879701; doi:10.3389/fneur.2022.936787)
Supplement: Supplementary file 1 [file Data_Sheet_1.PDF]

OBSERVATION CHECKLIST

Patient (PT) ID:	Caregiver (CG) ID:	Date:
Place:		
Psychiatric problem if any:	Communication problem if any:	Cognitive Problem if any:
NIH Score:		

**Relevance and comprehensibility of the intervention**		
**Does the stroke survivor have**	**Yes**	**No**
Needs related to information about stroke?	☐	☐
Needs related to rehabilitation of physical disabilities following stroke?	☐	☐
Needs related to performance of activities of daily living (ADL)?	☐	☐
Needs related to preparing self for daily living?	☐	☐
Needs related to information about assistive devices for his/her disabilities?	☐	☐
**Does the primary caregivers of the stroke survivor have**		
Needs related to information about stroke?	☐	☐
Needs related to training to care for a stroke survivor?	☐	☐
Needs related to enable the stroke survivor to independently do his/her ADL?	☐	☐
Needs related to enable the stroke survivor to prepare him/herself for ADL?	☐	☐
**Can the stroke survivor**		
Understand the instruction to operate the smartphone to access the intervention	☐	☐
Access the intervention relevant to his needs?	☐	☐
Read the directions and choices in the application?	☐	☐
View the pictures and access appropriate intervention content?	☐	☐
Operate the symbols/icons in the application to access the intervention?	☐	☐
Slide/scroll the screens for accessing the intervention content?	☐	☐

**Can the primary caregivers of the stroke survivor**		
Understand the instruction to operate the smartphone to access the intervention	☐	☐
Access the intervention relevant to his needs?	☐	☐
Read the directions and choices in the application?	☐	☐
View the pictures and access appropriate intervention content?	☐	☐
Operate the symbols/icons in the application to access the intervention?	☐	☐
Slide/scroll the screens for accessing the intervention content?	☐	☐
**Operational difficulties of the participants in using the smartphone**		
**Can the stroke survivor**	**Yes**	**No**
Hold the smartphone with affected hand and operate?	☐	☐
Hold the smartphone with unaffected hand and operate?	☐	☐
Hold the smartphone with both the hands and operate?	☐	☐
Pick up the smartphone and place it on a table top for operating or viewing?	☐	☐
Touch the smartphone screen, as and when required for viewing the visuals?	☐	☐
Slide the smartphone screen, as and when required for viewing the visuals?	☐	☐
View the written information, visuals, icons and videos of the intervention on the smartphone?	☐	☐
Hear the instructions on the videos clearly?	☐	☐
Access the intervention videos without any difficulties?	☐	☐
**Can the primary caregiver of the stroke survivor**		
Hold the smartphone with their hand and operate?	☐	☐
Hold the smartphone with both the hands and operate?	☐	☐
Pick up the smartphone and place it on a table top for operating or viewing?	☐	☐
Touch the smartphone screen, as and when required for viewing the visuals?	☐	☐
Slide the smartphone screen, as and when required for viewing the visuals?	☐	☐

View the written information, visuals, icons and videos of the intervention on the smartphone?	☐	☐
Hear the instructions on the videos clearly?	☐	☐
Access the intervention videos without any difficulties?	☐	☐
**User-friendliness of the intervention**		
	**Yes**	**No**
Is the smartphone too big to use?	☐	☐
Is the Smartphone too heavy to use?	☐	☐
Is there any difficulty in signing in to access the intervention?	☐	☐
Is there any difficulty in reading the texts in Tamil or English from the intervention pages?	☐	☐
Is the size of the texts / words in the intervention pages big enough to read?	☐	☐
Can the user make out the sections of the intervention from the pictures?	☐	☐
Are the intervention videos clear and audible?	☐	☐
Is the size of the Smartphone screen in wide enough to view the intervention videos clearly?	☐	☐
Can the user view the videos by operating the video icons correctly?	☐	☐
Can the user view any number of the videos any number of times they need?	☐	☐
Can the user view any sections of the videos as required by touching/sliding appropriate icons?	☐	☐
Can the user carry the intervention and view it wherever they go and whenever they need?	☐	☐
Can the user contact you for any queries through the contact options?	☐	☐

**Technical issues in the Application**									
**Application issues**	**Yes**	**No**	**Content Issues**	**Yes**	**No**	**Device Issues**	**Yes**	**No**	**Monitoring Issues**
Touch option	☐	☐	Uploading	☐	☐	Battery life	☐	☐	Report Capture
Slide option	☐	☐	Streaming	☐	☐	Charging time	☐	☐	Report Generation
Scrolling option	☐	☐	Viewing	☐	☐	Touch Screen damage	☐	☐	Report Monitoring
Signing in	☐	☐	Media-player default	☐	☐	Heat	☐	☐	
Page alignment	☐	☐	Picture clarity	☐	☐	Key/Access icon	☐	☐	

						issues			
Thumbnail generation	☐	☐	Sub-Title viewing	☐	☐	Program locking	☐	☐	

**Training needs in order to access the intervention**		
	**Yes**	**No**
Training required for stroke survivor?	☐	☐
Training required for primary Caregiver of stroke survivor?	☐	☐
Training Manual required?	☐	☐
