# Supplementary material for: Evaluation of the feasibility and acceptability of ReWin—A digital therapeutic rehabilitation innovation for people with stroke-related disabilities in India
Source: Front Neurol. 2023 Jan 12;13:936787. doi: 10.3389/fneur.2022.936787 (PMC9879701; doi:10.3389/fneur.2022.936787)
Supplement: Supplementary file 2 [file Data_Sheet_2.PDF]

Study Title: Development and Evaluation of Feasibility and acceptability of TNQ in Gage

Stroke Tele-Rehabilitation (TIST) Intervention in Tamilnadu India

Satisfaction Survey

Assessment form for participants

Participant Study ID: **Date of Assessment**

Orienting and training participants to the Intervention

1. What was your initial impression about an intervention like this? Please explain

2. When you were told that you would receive this intervention for 2 weeks, how did you feel?

3. To use this intervention, do you think that you need

   Training Support from caregivers Both Manage yourself Not sure

4. Did you receive sufficient information about the intervention before it was handed over to you?

   Yes, definitely Yes, to some extent No

5. Were the instructions provided to you to access the intervention from Smartphone or tab or computer clear and understandable?

   Yes, definitely Yes, to some extent No

6. Do you the think, the demonstration provided to you to access the intervention from Smartphone or tab or computer clear and understandable?

   Yes, definitely Yes, to some extent No

7. Do you think that a instruction booklet will be helpful to you to access the intervention?

   Yes, definitely Yes, to some extent No

8. Did you get sufficient opportunity to try accessing the intervention from a Smartphone or tab or computer yourself - before it was handed over to you?

   Yes, definitely Yes, to some extent No

9. Did you have enough confidence to try out this intervention when it was provided to you?

   Yes, definitely Yes, to a greater extent, Yes, to some extent Yes to a very small extent

   Not confident

10. Overall do you think you received sufficient training and support to access the intervention from the Smartphone or tab or computer?

    Yes, definitely Yes, to some extent No

Accessing the intervention

11. Did you access the intervention on the Smartphone or tab or computer by yourself? Yes / No

    If yes, go to question 12   if no, go to question 14

12. Was it easy to navigate between the pages and the intervention easily?

    Yes, definitely   Yes, to some extent   No

13. Did you have any difficulty in accessing the intervention from Smartphone or tab or computer ? Yes / No

    If yes, please mention the difficulties you experienced.

    ____

    ____

    ____

14. Who helped you to use the intervention? ______________________________

15. Was it easy for them to navigate between the pages and the intervention easily?

    Yes, definitely   Yes, to some extent   No

16. Did they have any difficulty in accessing the intervention? Yes / No

    If yes, please mention the difficulties that they experienced.

    ____

    ____

    ____

Content of the Intervention

17. Do you think that the video information was presented in a way you could watch and understand?

    Yes, Definitely   Yes, to some extent   No

18. Was the intervention relevant to the current needs?

    Yes, completely   Yes, to some extent   No

19. Which section was more interesting to the stroke survivor?

    Stroke information   Home-based exercises   Assistive devices   Functional skills   ADL   All   None

Utilisation of the Intervention

20. How do you feel about the length of time from being discharged from hospital to being given this intervention

    The intervention was given to us earlier than I thought was necessary

    The intervention was given to us as soon as I thought was necessary

    The intervention should have been given to us sooner

The intervention should have been given to us much sooner

21. How often did the stroke survivor use the intervention in the past two weeks?

    Once or more than once daily   Once or more than once weekly   Whenever necessary

    Whenever possible   Did not use

22. Was it only the stroke survivor, who watched the intervention?   yes / no

23. If no, please mention those who watched the intervention videos

    ____

24. Do you think that the intervention is useful to the stroke survivor?

    Yes, completely   Yes, to some extent   No

25. If yes, in what ways were the aspects useful? Please explain

    ____

    ____

    ____

26. Please mention three things that you liked most about this intervention

    ____

27. Please mention three things that you liked least about this intervention

    ____

28. Have you seen similar kind of intervention before?   Yes / No

29. If yes, was there anything new in this intervention – please explain?

    ____

    ____

30. Did the stroke survivor try doing some activities or exercises themselves or with the help of the family after using the intervention?

    Yes, always   Yes, frequently   Yes, Occasionally,   Yes, Rarely,   Never practice

    No

31. Do you think, two weeks is sufficient time given to the stroke survivor and you to use this intervention?

    Yes, definitely   yes, probably   No, I will need it for some more time

32. Will you support the stroke survivor in using this intervention even after you give the Smartphone or tab or computer back? Yes / No

33. If yes, how will you do that? Please explain

34. Overall, Did you like this intervention

Yes, definitely Yes, to a great extent Yes, to some extent No

35. Do you think this intervention would be useful for someone affected by stroke?

Yes, definitely Yes, probably No

36. How useful was this intervention?

Extremely useful Very useful Useful to an extent Not useful

37. How would you rate this intervention you received?

Excellent Very good Good Fair Poor

38. Would you recommend this intervention to your friends and family?

Yes, definitely Yes, probably No

39. If no, please comment

____

40. Other Comments / Suggestions
